# An Analysis of Post-Adrenalectomy Dynamics in MACS (Mild Autonomous Cortisol Secretion)-Positive Adrenal Tumours: The Biomarkers and Clinical Impact

**DOI:** 10.3390/jcm14155217

**Published:** 2025-07-23

**Authors:** Alexandra-Ioana Trandafir, Mara Carsote, Alexandru-Florin Florescu

**Affiliations:** 1 PhD Doctoral School, “Carol Davila” University of Medicine and Pharmacy, 020021 Bucharest, Romania; alexandra-ioana.trandafir@drd.umfcd.ro; 2 Department of Endocrinology, “Carol Davila” University of Medicine and Pharmacy, 020021 Bucharest, Romania; 3 Department of Clinical Endocrinology V, “C.I. Parhon” National Institute of Endocrinology, 011863 Bucharest, Romania; 4 Endocrinology Department, “Grigore T. Popa” University of Medicine and Pharmacy, 700111 Iasi, Romania; alexandru-florin.florescu@umfiasi.ro; 5 Endocrinology Department, “Sf. Spiridon” Emergency County Clinical Hospital, 700111 Iasi, Romania

**Keywords:** cortisol, marker, hormone, assay, detection, adrenalectomy, adrenal, tumour, surgery, ACTH, dexamethasone

## Abstract

**Background/Objective:** One third of “non-functioning adrenal tumours” (NFAs) have mild autonomous cortisol secretion (MACS). An updated analysis of the hormonal biomarkers profile, including risk factors and the rate of post-surgery adrenal insufficiency (PSAI), the duration of restoring the normal adrenocortical function in MACS/NFA and potential impacts on clinical comorbidities. **Methods:** Comprehensive review based on PubMed search (January 2020–January 2025). **Results:** The studies (n = 14) included 2623 patients (N = 1158 underwent unilateral adrenalectomy), aged 18–93 (mean = 57.49 years), with a female-to-male ratio = 1.54. Post-adrenalectomy (n = 9, N = 753) analysis: the PSAI risk correlated with the severity of baseline hypercortisolism. PSAI incidence: 50% of MAC. The rate after 4–6 weeks follow-up was 71.9% (adrenal Cushing’s syndrome) vs. 50% (MACS) vs. 14.4% (NFA). PSAI duration was up to 35 months. Early PSAI diagnosis was reflected by post-operative cortisol assay on day 1 (cut-off ≤ 5 µg/dL) and an ACTH (Cosyntropin) stimulation test (CST) (cortisol cut-off ≤ 14 µg/dL). Pre-operatory PSAI predictors: higher serum cortisol-DST (1 mg dexamethasone testing) and lower baseline plasma ACTH (not all studies agreed). **Conclusions:** A stratified strategy is encouraged following a unilateral adrenalectomy in MACS; PSAI is expected in almost half of patients, with a potential improvement of hypertension. Serum cortisol assays serve as most useful biomarker as pre-operatory PSAI predictor (after DST) and, potentially, in addition with baseline ACTH. Post-surgery basal cortisol measurement (± CST) helps the decision of glucocorticoids replacement since first post-operative day and during follow-up, serial testing at 3 months is a useful tool.

## 1. Introduction

The spectrum of endogenous persistent hypercortisolism varies, and it includes recently defined entities such as mild autonomous cortisol secretion (MACS) in adrenal tumours, characterized by hormonal changes in the hypothalamic–pituitary–adrenal (HPA) axis without overt Cushing’s syndrome (CS) [1,2,3]. The MACS-positive profile is typically associated with accidentally detected adrenal masses, namely, adrenal incidentalomas (AI), and often affects women of menopausal age [2,3].

While most AIs are hormonally non-functional, hence the term “non-functioning adrenal tumours” (NFAs) is generally used, actually, one third of them are MACS-positive [1,2,3]. The overall phenotype embraces a heterogeneous panel across cross-sectional and longitudinal analyses even in the same individual, and many aspects are still a matter of debate, including the clear hormonal assays to define MACS, the indications of adrenalectomy, and the potential changes in terms of post-surgery biomarkers and the clinical picture [4].

AI are diagnosed in 1% to 20% of adults depending on age. Most data have indicated that MACS-positive tumours have a high cardio-metabolic impact (e.g., hypertension, type 2 diabetes, dyslipidaemia) and increased associated risk of acute cardiovascular events [5,6,7], as well as an elevated prevalence of osteoporosis and fragility fractures [8,9,10]. Overall mortality is also increased in MACS-positive patients compared to controls according to some authors [11,12].

An adrenalectomy potentially improves the outcome and reverses some complications in patients [13,14,15]. Yet, this is not a mandatory part of management in all cases versus (vs.) the conservative approach. Of note, the risk of post-surgery chronic adrenal insufficiency should be taken into consideration in addition to the risk of an acute crisis developing under special circumstances [16,17,18]. However, the risk of this being a permanent consequence upon unilateral tumour removal is minor [19,20,21]. Glucocorticoid replacement is still challenging with regard to duration and regimes starting with the perioperative protocol [22,23,24], while withdrawal syndrome may be caused by a prior steroids overdose [25,26,27].

To summarize, a complex approach requires not only prompt MACS/NFA recognition, but also requires establishing an indication for adrenalectomy. Surgery may be followed by dynamic post-operative changes, including a cardio-metabolic and bone status improvement (or not) in addition to developing transitory adrenal insufficiency in cases with unilateral tumour removal [28,29,30]. Hence, it is important to address the issue of the post-surgery hormonal picture since only limited data has been published so far on this particular instance of MACS.

### Objective

We aimed to provide an updated analysis of the hormonal biomarkers profile, including the risk factors and the rate of developing postoperative adrenal insufficiency, as well as the duration of restoring the normal adrenocortical function in individuals confirmed with NFA/MACS and the potential impact of these post-adrenalectomy changes on (clinical) comorbidities.

## 2. Methods

**Figure 1:**
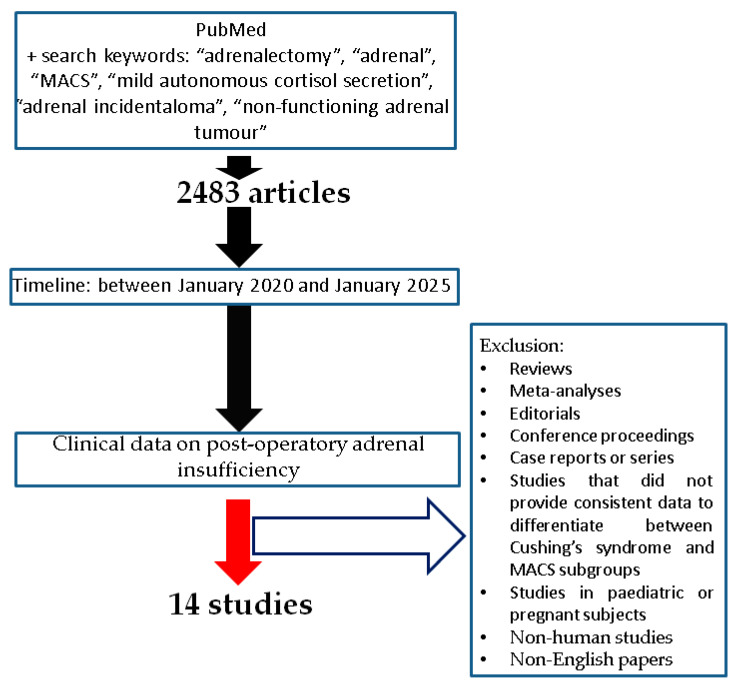
Flowchart of search according to our methods [31,32,33,34,35,36,37,38,39,40,41,42,43,44].

This comprehensive review was based on a literature search using the following criteria: full-length articles accessible via PubMed (English language) and subjects diagnosed with NFA/MACS who underwent an adrenalectomy. The search keywords were (in different combinations): “adrenalectomy”, “adrenal”, “MACS”, “mild autonomous cortisol secretion”, “adrenal incidentaloma”, “non-functioning adrenal tumour”. The search was limited to human studies with a publication date between January 2020 and January 2025. We included any type of study design and only the studies that provided biomarkers (e.g., hormonal panel) following surgery for unilateral adrenal tumour removal. Exclusion criteria were as follows: reviews, meta-analyses, editorials, conference proceedings, case reports, and studies that did not provide consistent data to differentiate between CS and MACS subgroups, and studies in pediatric or pregnant subjects [31,32,33,34,35,36,37,38,39,40,41,42,43,44] (Figure 1).

## 3. Results

**Figure 2:**
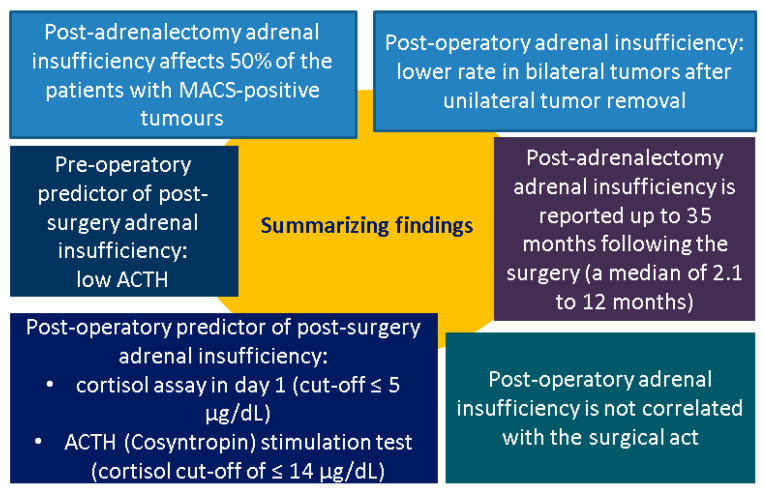
Summary of findings [31,32,33,34,35,36,37,38,39,40,41,42,43,44] (abbreviations: ACTH = adrenocorticotropic hormone; MACS = mild autonomous cortisol secretion).

The included studies (n = 14) had various designs: one was cross-sectional, three were prospective, three were retrospective, and seven used cohorts. A total of 2623 patients were included (female-to-male ratio of 1.54), aged between 18 and 93 years (mean age of 57.49 years), and 1158 of them underwent an adrenalectomy [31,32,33,34,35,36,37,38,39,40,41,42,43,44] (Table 1, Figure 2).

The MACS diagnosis criteria were different among the articles included, but most authors agreed on the blood cortisol cut-off of more than or equal to 1.8 µg/dL upon 1 mg dexamethasone testing (DST) [31,33,36,40] and a second cut-off of less than 5 µg/dL [42], without a full-blown CS phenotype. Alternatively, combined criteria associated the serum assays for adrenocorticotropic hormone (ACTH) and urinary free cortisol (UFC) [32]. Other authors used the term “possible autonomous cortisol secretion” (PACS) for a second-day plasma cortisol between 1.9 and 5 µg/dL after DST [35,37,39], which is currently assimilated into the “MACS” terminology (Table 2).

The primary endpoints of the included studies involved an evaluation of pre- vs. post-surgery status regarding the lab findings and clinical features (mostly, cardio-metabolic, osseous, and cognitive function) and a comparison between MACS-positive and MACS-negative tumour-associated behaviour following adrenalectomy or the surgical approach in NFA/MACS compared to a conservative management [31,32,33,34,35,36,37,38,39,40,41,42,43,44] (Table 3).

### 3.1. Hormonal Biomarkers upon Unilateral Adrenalectomy

There is still uncertainty regarding the postoperative course in MACS-positive patients, meaning we lack clear criteria for forestalling post-surgery adrenal insufficiency or improvement of cortisol-related clinical characteristics. Generally, the definitive cure for this type of tumour remains the adrenalectomy, with laparoscopy being the recommended technique due to the low perioperative mortality and morbidity rate, including decreased blood loss, shorter hospital stay, and fewer surgical complications than open adrenalectomy [45,46,47,48].

As mentioned, an important element upon performing a unilateral adrenalectomy in MACS/NFA patients is further developing an adrenal insufficiency which is expected to be transitory. HPA controls the synthesis of cortisol in both adrenal glands amid a negative feedback loop under normal physiological conditions. The contralateral adrenal gland is frequently suppressed in the setting of a (cortisol-producing) functioning adrenal adenoma, either due to atrophy or general HPA axis suppression. In MACS-positive tumours, the cortisol secretion is mild, but persistent; hence, it cannot be clearly anticipated if, after the tumour removal, a patient will develop adrenal insufficiency [49,50,51,52].

Clear data (according to our methods) on postoperative adrenal insufficiency were available in nine studies (N = 753) [32,33,34,35,36,38,39,40,41,42,43,44]. It should be noted that, the risk of developing it was correlated to the severity of hypercortisolism in MACS-positive tumours. The incidence was 50% (the highest of 67.4%), with glucocorticoids replacement being administrated for up to 35 months [31,32,33,34,35,36,37,38,39,40,41,42,43,44] (Table A1). Some studies included post-operative cortisol measurement on day 1 after adrenalectomy (POD1) and an ACTH stimulation test in order to identify the individuals who may require further steroid replacement early. For instance, a prospective study in 108 patients who underwent an adrenalectomy (N = 47 subjects had MACS-positive status) defined MACS as a cortisol level after DST > 1.8 μg/dL, while individuals with CS had a cortisol level ≥ 5 µg/dL. Cosyntropin (ACTH) stimulation testing (CST) on postoperative day 1 (POD1-CST) identified anomalies in 47% of all patients. Of the subjects with MACS-positive profile, 27/47 required glucocorticoids replacement that was initiated after detecting a post-surgery baseline plasma cortisol level of ≤5 µg/dL or an abnormal POD1-CST cortisol value of ≤14 µg/dL. One year after adrenalectomy, 19% of these prior MACS-positive individuals still required glucocorticoid replacement. The median length of post-operative steroid replacement was 2.1 months, with an interquartile interval (IQR) of 0.75–4.6 vs. patients with full-blown CS [of 6.0 (1.4–17.1) months]. Preoperative levels of cortisol after DST were statistically significant higher (11.1 vs. 2.8 µg/dL, *p* = 0.015) and baseline plasma ACTH was lower (0 vs. 2.25 pg/mL, *p* = 0.008) in patients that required glucocorticoid replacement for more than 12 months compared to those who needed the substitution for <3 months [36].

Another small sample-sized study in 35 patients with bilateral AIs who were either referred for unilateral adrenalectomy (N = 27) or conservatively managed (N = 8) found a rate of post-operative adrenal insufficiency of 25.9%. Surgery candidates had lower second day plasma cortisol levels following DST (1.1 vs. 2.9 µg/dL, *p* = 0.003) and higher baseline ACTH levels (20 vs. 6 pg/mL, *p* = 0.001) compared to those without an adrenalectomy [35].

On the other hand, the patients who underwent an adrenalectomy had a higher ACTH, lower UFC, but higher cortisol after 1 mg DST vs. non-surgery candidates according to another study in 55 patients. No perioperative complications were reported. CST was routinely used to evaluate the adrenal function. The rate of post-operative adrenal insufficiency was 40%, with a duration of 12.3 ± 9.0 (6–35) months and serial testing every 3 months to check that the function restored during this time [38].

Of important note, routinely prescribing glucocorticoid replacement for all patients who underwent a unilateral adrenalectomy may lead to over-prescription and potential negative effects, since not all patients with MACS-positive tumours display adrenal insufficiency. A stratified strategy is general; y used than routine steroids administration, an aspect which is still a matter of debate. For example, subjects with POD1 levels of ≤5 μg/dL or cortisol levels after CST of ≤14 μg/dL were found to be glucocorticoids candidates in another study (N = 207 patients, and 70 of them were MACS-positive). Adding dynamic testing (CST) instead of simply using the basal cortisol levels increased the detection rate for post-surgery adrenal insufficiency [42].

Another approach may be starting steroid substitution in each patient immediately after the surgical procedure and then (within two to three months) re-assessing the adrenal function to select those who will continue with the substitution. For instance, we mention the study by Eller-Vainicher et al. [32] that aimed to identify hypercortisolism in AIs patients using 1 mg DST, midnight salivary cortisol (MSC), UFC, and ACTH and then to predict the absence of hypocortisolism after surgery. In 60 patients who underwent tumour excision (laparoscopic or laparotomic adrenalectomy was decided depending on the tumour size), pre-operatory hypercortisolism was confirmed based on having ≥ 3 criteria out of the followings: plasma cortisol after 1 mg DST > 3 µg/dL, UFC > 60 mg/24 h, ACTH < 10 pg/mL, and MSC > 5.4 mg/dL. No patient had perioperative and postoperative surgical complications. Steroid replacement [hydrocortisone 100 mg intravenously and cortisone acetate orally, at weight-related dosing ranging between 25 and 37.5 mg/day in three subdivided doses per day, an equivalent to 20–30 mg/day hydrocortisone] was recommended for all patients to prevent hypoadrenalism. After 60 days, patients were tested using a low-dose CST following a 24 h steroid withdrawal. Cortisol cut-offs of 16 µg/dL and 22 µg/dL were applied to diagnose and rule out post-surgical hypocortisolism, respectively. Post-operative adrenal insufficiency was confirmed in 39/60 patients, and the lowest pre-surgery blood cortisol after DST was 1.2 µg/dL in this subgroup [32].

Another study on 260 patients (56.5% were females) who were followed up on for a median of 8.8 (2.0–20.8) years enrolled three subgroups based on the serum cortisol levels upon 1 mg DST: autonomous cortisol secretion (ACS) > 5.0 μg/dL, possible autonomous cortisol secretion (PACS) 1.9–5.0 μg/dL (which may be assimilated into MACS category according to the current nomenclature), and NFA with cortisol ≤ 1.8 μg/dL. 23.5% of them underwent adrenalectomy (71.7% had a laparoscopic surgery). A total of 20.4% of the surgery candidates developed postoperative complications (e.g., acute pancreatitis, allergic reactions, intestinal atony, hematoma, lymph fistula, pneumonia, and wound infections, etc.), while adrenal insufficiency was more common in individuals with PACS and ACS than NFA (42.9% vs. 64.3% vs. 4.5%, *p* < 0.001) [39].

A simple alternative that might not be applicable in every case was pointed out in a small study (N = 32 adrenalectomy candidates, aged between 49 and 71 years, 59.4% were females) that assessed post-surgery adrenal function without steroids replacement until day 6. At that point, cortisol assays confirmed a normal function only in 18.8% of them. Among the other 81.2% of the subjects with low plasma cortisol, the adrenal function of 53.8% was restored according to a 6-week assessment. A receiver operating characteristic (ROC) curve was used to identify a threshold for pre-surgery cortisol after the application of 1 mg DST of ≤4.7 µg/dL predicting a 6-week recovery with 89.5% sensitivity and 72.7% specificity [40]. The same subgroups (PACS, N = 68 vs. ACS, N = 53) were analyzed after the adrenalectomy and were found to have higher serum baseline cortisol (8.0 ± 5.7 vs. 5.0 ± 2.6 µg/dL; *p* = 0.03), a lower rate of glucocorticoid replacement (59% vs. 89%; *p* = 0.003), and a shorter steroids substitution duration (4.4 ± 3.8 vs. 10.7 ± 18.0 months; *p* = 0.04) [33]. Also, when compared to CS, as expected, the rate of post-surgery adrenal failure was lower in non-CS (CS vs. ACS vs. NFA: 72.8% vs. 59.5% vs. 10.5%), respectively, at 4- to 6-week follow-up of 71.9% vs. 50% vs. 14.4% [34]. On the contrary, Guo et al. [41] showed in 209 patients (36.3% were conservatively managed vs. 63.7% who were surgically managed, specifically, 7.3% of them had total adrenalectomy, and 92.7% had partial adrenalectomy) that none of them developed hypocortisolism after tumour removal [41]. This might suggest a true non-functional profile before the surgery or suboptimal testing for the post-operative impairment of adrenal function. Notably, in this study-based analysis [31,32,33,34,35,36,37,38,39,40,41,42,43,44], no postoperative death was reported.

### 3.2. Impact of Adrenalectomy on Cardio-Metabolic Status

Post-adrenalectomy biomarkers that highlight adrenal function are translated into the clinical picture, and an expected improvement in cardio-metabolic comorbidities might be connected to the correction of cortisol excess. Generally, the most prevalent ailment is arterial hypertension (and its complications), which is found in over 60% of the MACS-positive patients [53,54]. Many pathogenic mechanisms such as an increased mineralocorticoid activity, changes in the renin–angiotensin–aldosterone system, anomalies in the blood vessels regulatory system, vascular remodelling, enhanced neurotransmitters synthesis, and an increased vascular response to catecholamines contribute to high blood pressure under the circumstances of persistent cortisol excess [54]. Overt hypercortisolism or long-term exposure to exogenous glucocorticoids are linked to an elevated risk of type 2 diabetes mellitus, as well as insulin resistance via reducing insulin action and decreasing glucose disposal [55]. In subjects diagnosed with MACS-positive tumours, the prevalence of glucose profile anomalies varies, up to a maximum rate for type 2 diabetes of 40%, according to some data [56,57]. Dyslipidaemia of any type was observed in 55% to 71% of these individuals [58,59]. Cortisol anomalies may disrupt lipid metabolism, leading to elevated levels of low-density lipoprotein (LDL) cholesterol and triglycerides, which worsen atherosclerosis. In adipose tissue, glucorticosteroids serve a dual role in stimulating both lipogenesis and lipolysis, which might lead to dyslipidaemia and adipose tissue alterations, as well as visceral obesity [60,61].

Nevertheless, the majority of the studies in our sample suggested that adrenalectomy significantly improved prevalent cardio-metabolic elements [34,35,37,38,39,41,43,44] (Table A2). Yet, as specified, these studies (n = 8, N = 1182 subjects, mean age of 57.8 years, 1100 females and 782 males) had the disadvantage of a heterogeneous MACS definition and mixed cohorts (MACS with NFAs), on one hand, and, on the other, they had a relatively low duration of post-surgery follow-up (mostly, for one year). Four studies evaluating the high blood pressure showed a statistically significant post-adrenalectomy improvement in both groups of patients, with MACS-positive tumours or NFAs; another two cohorts addressed the potential beneficial effects on the glucose–metabolic profile, six studies provided data on lipid profile, and another three on obesity upon surgical management [31,32,33,34,35,36,37,38,39,40,41,42,43,44,45].

For instance, Morelli et al. [38] showed that the pre-surgery elevated blood pressure worsened in the conservative group, 26.7% vs. 4% in the adrenalectomy group (*p* = 0.03) during follow-up. The improvement in hypertension was independently associated with the surgical approach with an odds ratio (OR) of 3 (95% CI between 3.8 and 108.3, *p* < 0.001). The glucose profile worsened in 20% and 8% of the same subgroups, respectively, which was not statistically significant during follow-up (*p* = 0.12). Furthermore, the pre-operatory serum cortisol assay after the application of 1 mg DST was predictive for achieving post-surgery blood pressure control. The cut-off with the highest diagnosis accuracy was 2.7 µg/dL (77% sensitivity, and 75% specificity). This threshold may help the make the decision between surgery and conservative management in MACS/PACS tumours [38].

Further results displayed a heterogeneous landscape of outcomes regarding cardio-metabolic profile. A retrospective analysis enrolled 259 subjects with ACS (42 individuals in the surgical subgroup and another 217 in the control subgroup) and 486 patients with NFAs (16 in the surgical subgroup and 470 in the control subgroup). Baseline cortisol after DST was >1.8 µg/dL (ACS) and ≤1.8 µg/dL (NFA), respectively. Patients with NFA who underwent adrenalectomy had a better blood pressure control than the conservative group (*p* = 0.009), while other parameters such as the rate of patients who started an antihypertensive treatment (0% vs. 9.5%, *p* = 0.746), hypoglycaemic drugs (0% vs. 4.7%, *p* = 0.586), or lipid-lowering medication (16.7% vs. 13.6%, *p* = 0.833) were similar between the two subgroups. Additionally, the subjects with ACS who underwent an adrenalectomy showed a statistically significant decrease in triglycerides (*p* = 0.029) and blood glucose (*p* = 0.035) vs. conservative subgroup [37]. Another study found that the adrenalectomy group (N = 46) showed a statistically significant better weight (32.6% vs. 6.5%, *p* = 0.002), glycaemic level (45.7% vs. 15.2%, *p* = 0.002), and blood pressure control (45.7% vs. 23.9%, *p* = 0.029) vs. controls (N = 46) after a 48-month follow-up [43]. A longitudinal study in 271 subjects undergoing adrenalectomy (CS = 127, ACS = 45, NFA = 99) pinpointed that the use of medication against hypertension seemed to increase 1-year post-adrenalectomy in study groups with CS (*p* = 0.003) and NFA (*p* < 0.001), but then decreased over time. In the ACS group, the use of medication against hyperlipidaemia and diabetes decreased only slightly during follow-up [34].

The prevalence of arterial hypertension statistically significant decreased in surgery candidates vs. pre-operatory data (from 77% at diagnosis to 61.7% at follow-up, *p* < 0.05), but not obesity and dyslipidaemia [39]. At 1-year post-adrenalectomy, a remission of hypertension was displayed in 17.1% of the patients with NFA, a mild improvement in 40.7%, and no improvement in 41.1% of these individuals, according to Guo et al. [41].

On the contrary, the 1-year outcomes in the surgery (11 patients with MACS-positive tumours) vs. non-surgery (N = 6) groups were similar in Liu’s study [44], in terms of the overweight/obesity rate (54.5% vs. 50%, *p* = 1.000), impaired glucose profile/type 2 diabetes (45.5% vs. 33.3%, *p* = 1.000), arterial hypertension (45.5% vs. 66.7%, *p* = 0.620), and dyslipidaemia (63.6% vs. 83.3%, *p* = 0.600) [44]. Particularly for bilateral AIs (N = 35), one useful strategy for reducing cortisol excess amid MACS confirmation might be deciding on a unilateral adrenalectomy for the tumour with the largest diameter. However, the subgroup that was conservatively managed (N = 8) showed similar results to the adrenalectomy group (N = 27) regarding diabetes and hypertension improvement during follow-up [35].

### 3.3. Impact of Adrenalectomy on Bone Status

Bone density and quality are typically impacted in CS, but even in MACS-positive cases these aspects have been highlighted in some studies [62,63,64], particularly an increased vertebral fracture risk [65,66,67]. The study-focused analysis identified three studies regarding the assessment of post-surgery skeleton health and dynamics with a general low level of statistical evidence mostly due to a reduced sample size [31,34,44] (Table A3). Decreased levels of circulating osteocalcin as a bone formation marker showing an abnormal bone metabolism have been observed in subjects with MACS-positive tumours [31]. Athimulam et al. [31] conducted a study in 213 individuals with AIs [aged between 18 and 93 (median of 58) years; 67% were females]. A subgroup of these patients were analyzed regarding their pre- and post-surgery bone turnover markers profiles. Adrenalectomy-related changes in bone metabolism in MACS-positive subjects (N = 6) at a median of 37 (range 6–92) weeks revealed that osteocalcin and CTX (C-terminal telopeptide) statistically significantly improved; the mean differences were of 8.18 ng/mL (SD = 6.74, *p* = 0.04) and of 0.14 ng/mL (SD = 0.12, *p* = 0.05), respectively. PINP (type 1 procollagen amino terminal peptide) and sclerostin increased following adrenalectomy by 1.78 µg/L (SD = 35.4, *p* = 0.75) and 107 pg/mL (SD = 181, *p* = 0.24), but this was not statistically significant [31]. A different approach showed that subjects with CS and ACS required a decrease in/stop of anti-osteoporosis medication during the first 2-year post-surgery; however, this difference was not statistically significant (*p* = 0.119, *p* = 0.336) [34]. Another study reported similar results between conservative (N = 6) and surgical (N = 11) management in patients with MACS with respect to the prevalence of osteopenia/osteoporosis (63.6% vs. 50%, *p* = 0.644) after 12-month follow-up [44].

### 3.4. Cognitive Impairement and Quality of Life

Additionally, chronic hypercortisolemia may cause a cognitive impairment, hence reducing the quality of life, mostly in CS, but, also, in MACS-positive tumours with a rate higher than found in NFAs. For instance, patients with MACS have been reported to have elevated scores on the Beck Depressive Inventory (which assesses depressive symptoms) and worse scores on the Short Form-36 survey for mental and physical components vs. controls [68,69,70]. Insomnia and other sleep disorders have been found more often to have a relationship with MACS diagnosis by some authors [71]. Furthermore, a recent population-based study identified that subjects with AIs had higher rates of depression and anxiety vs. controls [72].

According to our analysis, two studies [34,44] pointed out the impact of surgical vs. conservative management on the cognitive performance in MACS-positive patients (Table A4). Liu et al. confirmed a cognitive impairment in MACS-positive subjects, while individuals with MACS and CS showed a comparable impairment. Remission of hypercortisolemia after surgery might improve the cognitive function. The study included 59 patients with NFA (mean age of 45.9 ± 10.4 years), 36 subjects with MACS (average age of 48.0 ± 8.5 years), and 20 individuals with adrenal CS (mean age of 41.9 ± 12.1 years). The logistic regression revealed that individuals with MACS (OR of 3.738, 95% CI: 1.329–10.515, *p* = 0.012) and CS (OR of 6.026, 95% CI: 1.411–25.730, *p* = 0.015) had a higher risk of immediate memory impairment. A 12-month post-adrenalectomy assessment pointed out that the cognitive function statistically significant improved (*p* = 0.035), according to Repeatable Battery for the Assessment of Neuropsychological Status scores [44]. Further more, in another study, the use of medication for depressive disorders decreased among ACS patients following adrenalectomy (*p* = 0.043) for an average follow-up of 2.6 years [34].

## 4. Discussion

### 4.1. MACS: From General Health Aspects to Various Biomarkers

This sample-based analysis pinpointed a large area of clinical and lab issues starting from the hormonal panel of biomarkers pre- and post-adrenalectomy [31,32,33,34,35,36,37,38,39,40,41,42,43,44].

While the topic of surgical management in MACS has been extensively explored in recent years, these studies have largely focused on clinical outcomes such as cardiovascular and metabolic parameters, quality of life, and risk stratification. By contrast, the present analysis is focused on the early post-operative dynamics of specific hormonal biomarkers, which are not the primary focus of previous reviews/meta-analyses. This biochemical perspective offers potentially useful insights into post-surgical monitoring and decision-making which is essential in the everyday practice. Hence, the original contribution involves an assessment of the detailed post-operative hormonal dynamics of three specific biomarkers (ACTH, cortisol, and associated dynamic testing), which are not the primary focus of previously published reviews.

Globally, MACS represents a widely discussed topic because of its rising prevalence/incidence. Over the years, numerous diagnosis criteria have been applied, but this is still a matter of debate [73,74,75]. Additionally, steroid metabolomics might be part of future diagnosis algorithms in many endocrine and non-endocrine ailments, serving as prognostic factors and elements in a multimodal tailored decision [76,77,78,79,80]. Steroid biomarkers might reveal different degrees of androgen suppression and glucocorticoid excess or, on the contrary, adrenal failure with a life-threatening need for replacement in some cases [81,82,83].

The studies (n = 14) included 2623 patients (N = 1158 of them underwent unilateral adrenalectomy), aged between 18 and 93 years with a female-to-male ratio of 1.54. Post-adrenalectomy (n = 9, N = 753) lab findings pointed out that the risk of post-surgery adrenal insufficiency correlated with the severity of baseline hypercortisolism [31,32,33,34,35,36,37,38,39,40,41,42,43,44]. Previously known as “subclinical hypercortisolism,” “subclinical CS”, or “preclinical CS”, MACS involves an excess of cortisol without the typical clinical manifestations of overt CS. The latest guidelines (from 2023) recommended diagnosis using 1 mg DST (a blood cortisol > 1.8 µg/dL is consistent with a MACS-positive feature) [84]. This explains why this 5-year analysis of previously published data introduced a rather inhomogeneous spectrum of diagnosis criteria for the adrenal tumours [31,32,33,34,35,36,37,38,39,40,41,42,43,44].

While we found no distinct genetic data [31,32,33,34,35,36,37,38,39,40,41,42,43,44] across the mentioned studies, on a deeper level, sporadic unilateral NFAs and MACS-positive adrenal tumours present similar genomic and transcriptome characteristics, distinguishing them from adrenal CS [85,86,87], with the most prevalent genetic aberration being a somatic *CTNNB1* pathogenic variant that activates the Wnt-β–catenin pathway [87,88]. Wnt-β–catenin signalling is essential for the growth of the adrenal cortex (as found in other tissues and organs), and it harbours a well-established involvement in the adrenal carcinogenesis via decreased apoptosis and enhanced cell proliferation [89]. Additionally, genetic testing for germline *ARMC5* pathogenic variants should be considered in individuals with multiple adrenal nodules and ACTH-independent MACS-positive profile, since benign adrenocortical nodular disease might present MACS features [90,91]. On the other hand, insulin resistance, hyperinsulinemia, and hypertension may supplementarily contribute to the development of an adrenocortical adenoma or hyperplasia, leading to a vicious cycle of cortisol excess and metabolic anomalies [92,93,94].

As mentioned, the analyzed cohort presented a female predominance with an overall mean age of 57.49 years [31,32,33,34,35,36,37,38,39,40,41,42,43,44]. Notably, most women with MACS are post-menopausal and, hence, they have high luteinizing hormone (LH) levels; some authors even suggested a potential contribution of LH to the adrenal tumorigenesis. It is noteworthy that insulin resistance in patients with metabolic syndrome and adrenal tumorigenesis positively correlated with the tumour diameter according to previous studies [95,96], while in this analysis we found no consistent data to correlate the laboratory assays to the tumour size at imaging evaluation [31,32,33,34,35,36,37,38,39,40,41,42,43,44]. Moreover, changes in the vascular supply to the adrenal cortex due to atherosclerosis or hypertension might cause local hypoxia and a compensatory cell proliferation [97].

A more complex view includes the fact that increased cortisol secretion was linked to a dysregulation of bioactive lipid classes such as lysoglycerophospholipids, ceramides, and sphingolipids, which regulate the cell cycle, immunomodulation, angiogenesis, intracellular trafficking, insulin signalling, and endothelial function [98,99]. Abnormal lipid-dependent cell signalling was connected to various chronic inflammatory diseases, e.g., obesity, type 2 of diabetes, non-alcoholic fatty liver disease, cardiovascular events, and even some malignancies [100,101,102]. Dyslipidaemia and its potential improvement upon tumour removal was analyzed at a low level of statistical significance in the mentioned studies [31,32,33,34,35,36,37,38,39,40,41,42,43,44]. On a practical level, overlapping circumstances due to age or even menopause-related hypo-estrogenic status might bias the interpretation of the lipids profile in these patients.

Two other important biomarkers should be noted in the field of MACS (they were out of our scope but worth mentioning). One is represented by chronic low-grade inflammation that remains a key pathogenic process. Cortisol modulates inflammation and immune system function, contributing to CS-related comorbidities like diabetes, visceral adiposity, atherosclerosis, osteoporosis, and cognitive impairment [103]. Moreover, elevated cortisol levels in MACS-positive tumours might induce a systemic inflammatory response, as reflected by elevated inflammatory markers such as C-reactive protein and interleukin 6. Long-term inflammation impairs the endothelial function and causes arterial stiffness, increasing the cardiovascular risk [104,105]. Secondarily, 10% to 30% of the individuals with primary aldosteronism have been found to display both cortisol hypersecretion (usually, MACS, not CS) and aldosterone excess (also known as “Connshing syndrome”). Hypertension is more common in these patients (OR of 7.7, 95% CI: 2.64–22.32) than in individuals with MACS-positive tumours (without aldosterone excess) [106]. Plasma aldosterone seems more important to the pathophysiology of hypertension in these subjects than in those with overt CS [107,108].

### 4.2. General Management: From Surgery to Post-Operative Medication

Generally, MACS-positive adrenal tumours-related management remains a challenge, with adrenalectomy being the preferred treatment as opposed to clear NFAs (MACS-negative patients) where a conservative approach is provided, with surgery being reserved for a selective subgroup. The decision should take into consideration HPA abnormalities, cortisol-related complications, and end-organ damage. Although postoperative biochemical testing frequently reveals abnormalities consistent with an adrenal insufficiency, their associated clinical significance and if low serum cortisol measurements and/or a blunted response to ACTH stimulation should pinpoint starting glucocorticoids replacement, or whether these patients can be safely followed without steroids exposure if they do not exhibit clear symptoms/signs of an adrenal failure are unclear [109,110].

Glucocorticoids substitution is advised for MACS patients with low preoperative basal ACTH levels, no response to corticotropin-releasing hormone load testing, and diminished-to-absent uptake in the contralateral adrenal gland on adrenal scintigraphy [111,112]. The current sample-based analysis based on the surgical management in MACS was centred on the identification of post-adrenalectomy adrenal insufficiency. This event was found with the following incidence rates: approximately 50% of MACS-positive patients (highest of 67.4% and lowest of 0% in one study, respectively, and 25% in bilateral NFAs/MACS); alternatively, the rate after 4 to 6 weeks of follow-up was 71.9% (CS) vs. 50% (MACS) vs. 14.4% (NFA). Post-surgery adrenal insufficiency duration occurred for up to 35 months; one study showed a median (IQR) of 2.1 (0.75–4.6) months; another found a mean ± SD of 12.3 ± 9.0 months; and another cohort showed that, after one year since tumour removal, 19% of MACS-positive subjects still needed steroids replacement. Generally, post-surgery status for CS is expected to require steroid substitution for up to 6–24 months [46,113].

Early diagnosis after surgery was supported by a post-operative cortisol assay on day 1 (cut-off ≤ 5 µg/dL) and an ACTH (Cosyntropin) stimulation test (cortisol cut-off of ≤14 µg/dL). Pre-operatory predictors were represented by higher serum cortisol after DST and lower baseline plasma ACTH (but not all studies agreed); one study identified a cortisol (after DST) cut-off ≤ 4.7 µg/dL that predicted 6-week recovery (89.5% sensitivity and 72.7% specificity), and another study showed the lowest cortisol level after DST of 1.2 µg/dL in patients who further developed adrenal insufficiency following tumour removal (66.6% accuracy) [31,32,33,34,35,36,37,38,39,40,41,42,43,44].

In most studies, the glucocorticoids replacement strategy showed that routinely testing all patients on day 1 (baseline cortisol ± CST) and then deciding the level of substitution based on cortisol cut-offs might be preferable. One study showed that starting steroids on day 0 and then re-checking (CST) at two months might indicate further need for replacement (a cortisol cut-off > 22 µg/dL ruled out an adrenal insufficiency, while a cut-off of <16 µg/dL confirmed it). Of note, during glucocorticoids replacement, serial testing at three months (e.g., 24 h glucocorticoids withdrawn followed by CST) helped with the substitution decision [31,32,33,34,35,36,37,38,39,40,41,42,43,44].

As mentioned, despite limited data, bilateral tumours require a more complex approach, including genetic testing or adrenal vein sampling to indicate the left–right gradient between the hormones of the adrenal cortex, and thus, to sustain the indication for surgery [114,115]. The presence of a bilateral secretion might explain the lower rate of adrenal insufficiency following a single adrenal gland removal, as shown in the study conducted by Yilmaz et al. [35].

Most procedures were laparoscopically performed since, nowadays, a minimally invasive adrenalectomy is a safe and successful therapeutic approach, including in patients with MACS [116,117,118]. The spectrum of the complications related to the operation, despite being rare, might correlate with numerous factors such as body mass index, patient’s age and comorbidities, tumour size, and malignant features, surgeon’ experience, etc. [50,119]. While persistent hypercortisolemia may impair the coagulation status, the vessels dynamics, glucose profile, and increased the risk of embolic events, we found no significant associations between post-surgery outcome of adrenal function and the surgical procedure itself [31,32,33,34,35,36,37,38,39,40,41,42,43,44]. Generally, the operation for MACS-positive tumours remains similar to other endocrine ailments, except for providing the peri-operatory hydrocortisone replacement [120,121,122,123,124,125,126,127,128].

### 4.3. Current Limitations and Future Research

As limitations of the current analysis, we mention the narrative design of the presentation as a single database search, but we intended to cover a large area of lab and clinical issues and not restrain ourselves to a systematic approach since the studies’ protocols largely varied.

With respect to the topic at hand, future controlled longitudinal studies on bone turnover markers and even cardio-metabolic (including glucose and lipids profile) markers are needed for a longer period of time. The cut-offs for baseline ACTH before surgery that are required to pinpoint MACS and to further (post-operative) indicate the need for glucocorticoids replacement are still debatable. At this point, ACTH measurement does not represent a standard criterion for MACS diagnosis. Similarly, the routine use of CST is rather depending on a local protocol than on a general standard care. Moreover, if a distinct subgroup of MACS-positive tumours is at higher risk of developing adrenal insufficiency is still an open issue, and we need models and algorithms of risk prediction for practical multidisciplinary purposes. Additionally, developing adrenal insufficiency might be regarded as a good prognostic factor for the outcome of previous cardiovascular and metabolic complications, but further correlational studies are necessary. Of note, there are only limited studies that primarily address the issue of post-surgery hormonal testing and this remains an ongoing issue. Moreover, there is a lack of standard post-operative protocols; for example, if replacement should be started in every patient or if a “wait and see” approach is more useful is still debatable. Also, routine dynamics tests following adrenalectomy are not generally applicable at this point in many centres.

## 5. Conclusions

- A stratified strategy is encouraged for post-unilateral adrenalectomy in MACS, while post-operative adrenal insufficiency should be expected in almost half of the patients.
- A potential long-term improvement in hypertension, and even the glucose profile, is expected after tumour removal.
- Patients with bilateral adrenal tumours who are referred for unilateral adrenalectomy display a lower risk of adrenal insufficiency, but, in this distinct instance, additional imaging surveillance of the adrenal tumour is required, as well as considering genetic testing.
- Serum cortisol assays serve as the most useful biomarker as a pre-operatory predictor of adrenal post-surgery function (specifically, amid DST, and, potentially, in addition to baseline ACTH assessment). Post-surgery basal cortisol ( ± CST) helps inform the decision to employ glucocorticoids replacement from the first post-operative day and during follow-up. Serial testing every 3 months is a useful tool for up to 35 months post-surgery.
- Routinely prescribing glucocorticoid replacement for all patients who underwent a unilateral adrenalectomy may lead to over-prescription and potential negative effects, since not all MACS patients display post-adrenalectomy adrenal insufficiency. Hence, a personalized strategy is encouraged.
- The operation does not seem to be correlated with the post-surgery outcome regarding the biomarkers, including endocrine profile, cardio-metabolic, and osseous outcomes.

## Figures and Tables

**Table 1:** Included studies that provided post-surgery data in patients with NFA/MACS tumours according to our methods (the display starts with 2020 until 2025) [31,32,33,34,35,36,37,38,39,40,41,42,43,44].

First Author Year of Publication Reference	Study Design	Number of Patients (Study Population) Sex Ratio (F/M) Age (Years)
2020 [31]	Cross-sectional, prospective study	**N = 213 patients with adrenal adenomas**; F/M = 143/7; age [mean (ranges)] = 58 (18–93) y **N1 = 22 patients with CS**; F/M = 18/4; age [mean (ranges)] = 41.5 (18–61) y **N2 = 92 patients with MACS**; F/M = 57/35; age [mean (ranges)] = 41.5 (18–61) y **N3 = 99 patients with NFA**; F/M = 67/32; age [mean (ranges)] = 59 (28–93) y **Post-adrenalectomy** **N1**′ **= 2 patients with CS** **N2**′ **= 6 patients with MACS** **N3**′ **= 8 patients with NFA**
2020 [32]	Cohort study	**N = 60 patients with AI who underwent adrenalectomy** (31 patients for adenoma size > 4 cm, 29 patients for presence of hypercortisolism) **N1 = 39 patients with postsurgical hypocortisolism**; F/M = 31/8; age [mean ± SD (ranges)] = 55.3 ± 12.0 (24–74) y **N2 = 21 patients without postsurgical hypocortisolism**; F/M = 15/6; age [mean ± SD (ranges)] = 57.2 ± 8.3 (33–75) y
2021 [33]	Retrospective study	**N = 121 patients who underwent adrenalectomy** **N1 = 68** patients with 1 mg DST cortisol 1.8–5 µg/dL; of these 56 had indeterminate nodule which included MACS F/M = 43/25; age (mean ± SD) = 58.5 ± 11.3 y **N2 = 53** patients with 1 mg DST cortisol > 5 µg/dL; of these 30 had indeterminate nodule which includes MACS F/M = 43/10; age (mean ± SD) = 52.4 ± 15.8 y
2021 [34]	Cohort study	**N = 271 with adrenal adenomas undergoing adrenalectomy** **N1 = 127 patients with CS**; F/M = 104/23; age (mean ± SD) = 56.9 ±12.6 y N2 = 45 patients with ACS *; F/M = 31/14; age (mean ± SD) = 65.0 ± 10.4 y **N3 = 99 with NFA**;F/M = 59/40; age (mean ± SD) = 60.5 ± 12.1y
2021 [35]	Retrospective study	**N = 35 patients with bilateral adenoma with subclinical hypercortisolemia** **N1 = 27 patients with unilateral adrenalectomy**, of these 21 had PACS * and 6 ACS *; F/M = 22/5; age [mean (range)] = 57 (34–75) y **N2 = 8 patients with conservative management**, of these 7 had PACS * and 1 ACS *; F/M = 4/4; age [mean (range)] = 65 (46–75) y
2022 [36]	Prospective cohort	**N = 108 patients who underwent adrenalectomy** **N1 = 47 patients with MACS**; F/M = 38/9; age [mean (range)] = 59 (54–66) y **N2 = 27 patients with CS**; F/M = 26/1; age [mean (range)] = 55 (45–60) y **N3 = 22 patients with PA**; F/M = 6/16; age [mean (range)] = 51 (38–55) y N4 = 12 with both PA/CS *; F/M = 4/8; age [mean (range)] = 58 (44–65) y
2022 [37]	Retrospective study	**N = 745 patients with adrenal adenomas** **N1 = 486 patients with NFA**; F/M = 273/213; age (mean ± SD) = 62.6 ± 10.72 y **N2 = 259 patients** (47 with confirmed ACS * and 212 with PACS *); F/M = 151/108; age (mean ± SD) = 64.7 ± 10.50 y **N1**′ **= 16 patients with adrenalectomy** **N1**″ **= 470 patients with conservative management** **N2**′ **= 42 patients with adrenalectomy** **N2**″ **= 217 patients with conservative management**
2022 [38]	Cohort study	**N = 55 AI patients with PACS** 6-month follow-up: **N1 = 25 patients with adrenalectomy**; F/M =17/8; age [mean (range)] = 63.2 ± 10.3 (41–76) y **N2 = 30 patients with conservative management**; F/M = 22/8; age [mean (range)] = 66.5 ± 9.1 (42–76) y
2023 [39]	Cohort study	**N = 260 patients adrenal adenomas**; F/M = 147/113; age [mean (range)] = 59.5 (20–82) y **N1 = 123 patients with NFA**; F/M = 69/54; age (mean ± SD) = 57 ± 62 y N2 = 96 patients with PACS *; F/M = 53/43; age (mean ± SD) = 63 ± 48 y N3 = 41 patients with ACS *; F/M = 25/16; age (mean ± SD) = 56 ± 51 y Post-adrenalectomy: **N**’ **= 61 patients** **N1**′ **= 22 patients with NFA** **N2**′ = 23 patients with PACS * **N3**′ = 16 patients with ACS * Conservative management: **N**″ **= 199 patients** **N1**″ **= 101 patients with NFA** **N2**″ = 73 patients with PACS * **N3**″ = 25 patients with ACS *
2023 [40]	Retrospective cohort study	**N = 32 patients with AI undergoing unilateral adrenalectomy**; F/M = 19/13; age [mean (range)] = 61 (51–66) y **N1 = 7 patients with NFA**; F/M = 5/2; age [mean (range)] = 66 (64–67) y **N2 = 25 patients with MACS**; F/M =14/11; age [mean (range)] = 58 (49–66) y
2024 [41]	Cohort study	**N = 309 patients with NFA**; F/M = 138/171; age (mean ± SD) = 54.9 ± 10.1 y **N1 = 123 patients with adrenalectomy**; F/M = 54/69; age (mean ± SD) = 54.2 ± 10.5 y **N2 = 186 patients with conservative management**; F/M = 84/102; age (mean ± SD) = 55.3 ± 9.8 y
2024 [42]	Cohort study	**N = 207 patients who underwent adrenalectomy**; F/M = 126/81; age [mean (range)] = 54.7 (45.9 –63.2) y **N1 = 42 patients with CS**; F/M = 39/3; age [mean (range)] = 52.0 (44.5–58.8) y **N2 = 70 patients with MACS**; F/M = 57/13; age [mean (range)] = 59.4 (51.9–68.2) y **N3 = 22 patients with mixed PA/hypercortisolism** *; F/M = 8/14; age [mean (range)] = 63.6 (53.7–65.9) y **N4 = 73 patients with PA**; F/M = 22/51; age [mean (range)] = 50.9 (40.3–57.9) y
2024 [43]	Prospective, multicenter study randomized	**N = 92 patients with MACS** **N1 = 46 patients with adrenalectomy**; F/M = 29/17; age (mean ± SD) = 53.6 ± 8.1 y **N2 = 46 patients with conservative management**; F/M = 29/17; age (mean ± SD) = 54.0 ± 7.4 y
2024 [44]	Prospective study	**N = 115 patients with AI** **N1 = 59 patients with NFA**; F/M = 31/28; age (mean ± SD) = 45.9 ± 10.4 y **N2 = 36 patients with MACS**; F/M = 25/11; age (mean ± SD) = 48.0 ± 8.5 y **N3 = 20 patients with CS**; F/M = 18/2; age (mean ± SD) = 41.9 ± 12.1 y **N2′ = 11 patients with adrenalectomy** **N2**″ **= 6 conservative management**

Abbreviations: AI = adrenal incidentaloma; ACS = autonomous cortisol secretion (* the designation was kept according to the original study); CS = Cushing’s syndrome; F/M = female-to-male ratio; MACS = mild autonomous cortisol secretion; N = number of patients; NFA = non-functioning adrenal tumours; PA = primary aldosteronism; PACS = possible autonomous cortisol secretion; SD = standard deviation; y = years (red font mean surgery candidates; **bold font describes the number of patients per subgroup**).

**Table 2:** The criteria for defining MACS/NFA within the analyzed studies [31,32,33,34,35,36,37,38,39,40,41,42,43,44].

Reference	Criteria for MACS/NFA Diagnosis
[31]	MACS: 1 mg DST cortisol > 1.8 µg/dL
[32]	Presence of hypercortisolism ≥ 3 out of: 1 mg DST cortisol > 3 µg/dL UFC > 60 mg/24 h ACTH < 10 pg/mL MSC > 5.4 mg/dL Postsurgical hypocortisolism (PSH) diagnosis and rule-out involve cut-offs of 16 µg/dL and 22 µg/dL.
[33]	MACS: 1 mg DST cortisol > 1.8 µg/dL
[34]	NFA: 1 mg DST cortisol ≤ 1.8 µg/dL ACS: 1 mg DST cortisol > 5 µg/dL, without clinical signs of CS
[35]	ACS: 1 mg DST cortisol > 5 µg/dL PACS: 1 mg DST cortisol: 1.9–5 µg/dL
[36]	MACS: 1 mg DST cortisol > 1.8 µg/dL CS: 1 mg DST cortisol ≥ 5 µg/dL PA: elevated plasma aldosterone, suppressed plasma renin activity, and aldosterone/renin ratio > 20, confirmatory testing with oral sodium loading or saline infusion test Abnormal POD1-CST: cortisol ≤ 14 (≤18 prior to April 2017).
[37]	NFA: 1 mg DST cortisol < 1.8 µg/dL PACS: 1 mg DST cortisol 1.9–5 µg/dL ACS: 1 mg DST cortisol > 5 µg/dL
[38]	PACS: 1 mg DST cortisol 1.9–5 µg/dL
[39]	NFA: 1 mg DST ≤ 1.8 µg/dL PACS: 1 mg DST 1.9–5 µg/dL ACS: 1 mg DST > 5 µg/dL
[40]	NFA: 1 mg DST cortisol ≤ 1.8 µg/dL MACS: 1 mg DST cortisol ≥ 1.8 µg/dL
[41]	NFA: 1 mg DST cortisol ≤ 1.8 µg/dL
[42]	CS: 1 mg DST > 5 µg/dL MACS:1 mg DST cortisol 1.8–4.9 μg/dL PA: increased plasma aldosterone levels, decreased plasma renin activity aldosterone/renin ratio > 20 Secondary adrenal insufficiency: POD1 basal cortisol level of ≤5 μg/dL, or a cortisol level of ≤14 μg/dL 60 min after CST Abnormal CST based on POD1 basal cortisol levels at thresholds between 5 and 15 μg/dL
[43]	MACS: 1 mg DST cortisol > 1.8 µg/dL
[44]	NFA: 1 mg DST ≤ 1.8 µg/dL MACS: 1 mg DST > 1.8 µg/dL CS: LDDST serum cortisol ≥ 1.8 µg/dL, elevated MSC and 24 h UFC, suppressed morning plasma ACTH

Abbreviations: ACTH = adrenocorticotropic hormone; ACS = autonomous cortisol secretion; CS = Cushing’s syndrome; CST = cosyntropin stimulation test; DST = dexamethasone suppression test; MACS = mild autonomous cortisol secretion; LDDST= low dose dexamethasone suppression test; MSC = midnight serum cortisol; NFA = non-functioning adrenal tumours; PSH = postsurgical (unilateral adrenalectomy) hypocortisolism; PACS = possible autonomous cortisol secretion; POD1 = postoperative day 1 cortisol; UFC = urinary free cortisol (the studies’ subgroups has been introduced in Table 1; green font = distinct subgroup with the designation of “MACS”; magenta = distinct subgroup with the designation of “PACS”).

**Table 3:** The outcomes analyzed in the studies’ patients [31,32,33,34,35,36,37,38,39,40,41,42,43,44].

Reference	Analyzed Outcomes
[31]	■ Bone turnover markers (BTMs) were compared between subjects with CS vs. MACS vs. NFA ■ In a subgroup of subjects, BTMs were compared before vs. after adrenalectomy
[32]	■ The study compared subjects with versus without PSH to detect hypercortisolism in AI patients using cut-offs such as 1 mg DST, MSC, UFC, and ACTH to predict the absence of post-surgery hypocortisolism ■ Analysis of the perioperative status
[33]	■ The study compared patients with preoperative DST of 1.8–5 µg/dL to patients with post-testing cortisol above 5 µg/dL, to a certain degree of postoperative glucocorticoid replacement necessary for patients undergoing adrenalectomy
[34]	■ Differences in drug use for depression, osteoporosis, hypertension, hyperlipidaemia, and anti-diabetic medications one year before vs. 1-year following adrenalectomy in subjects with CS, ACS, and NFA ■ Analysis of pre- and perioperative characteristics and surgical outcome in patients with adrenal tumours
[40]	■ Assessment of postoperative adrenal function
[41]	■ The study aimed to investigate the effect of adrenalectomy on hypertension in NFA
[43]	■ This study aimed to assess the metabolic impact of adrenalectomy vs. conservative approach in MACS
[44]	■ Cognitive function, metabolic comorbidities, and bone health were compared in subjects with CS, MACS, and NFA, as well as the effect of surgical versus conservative management in subjects with MACS ■ Analysis of the perioperative status

Abbreviations: AI = adrenal incidentalomas; ACS = autonomous cortisol secretion; ACTH = adrenocorticotropic hormone; BTM = bone turnover markers; CS = Cushing’s syndrome; DST = dexamethasone suppression test; MACS = mild autonomous cortisol secretion; MSC = midnight serum cortisol; NFA = non-functioning adrenal tumours; PSH = postsurgical (unilateral adrenalectomy) hypocortisolism; UFC= urinary free cortisol; vs. = versus (the studies’ subgroups has been introduced in Table 1).

## Data Availability

Not applicable.

## Abbreviations

The following abbreviations are used in this manuscript:

AI	adrenal incidentalomas
ACTH	adrenocorticotropic hormone
ACS	autonomous cortisol secretion
CS	Cushing’s syndrome
CTX	C-terminal telopeptide
CI	confidence interval
CST	cosyntropin stimulation test
DST	dexamethasone suppression test
DDD	defined daily dose
F/M	female-to-male ratio
IQR	interquartile interval
HPA	hypothalamic–pituitary–adrenal
LDDST	low dose dexamethasone suppression test
LDL	low-density lipoprotein
LH	luteinizing hormone
MACS	mild autonomous cortisol secretion
MSC	midnight serum cortisol
NFA	non-functioning adrenal tumours
N	number of patients
NA	not available
OR	odds ratio
PA	primary aldosteronism
P1NP	type 1 procollagen amino terminal peptide
PACS	possible autonomous cortisol secretion
PSH	postsurgical (unilateral adrenalectomy) hypocortisolism
POD1	postoperative day 1 cortisol
ROC	receiver operating characteristic
SD	standard deviation
y	years
UFC	urinary free cortisol
vs.	versus

## Appendix A

**Table A1:** Post-operative assessment in the study population: hormonal biomarkers [32,33,34,35,36,38,39,40,41,42,43,44].

Reference	Perioperative Characteristics	Perioperative Hormonal Assessment
[32]	Laparoscopic or laparotomic adrenalectomy was decided depending on the size (N = 31 for adenoma size > 4 cm and N = 29 for evidence of hypercortisolism) There were no perioperative complications following surgery N1 vs. N2 Hydrocortisone dose during substitutive therapy period (mg/day): 20.9 ± 2.6 vs. 20.9 ± 2.3, *p* = 0.932 Hydrocortisone replacement = all patients Hydrocortisone dose = 100 mg intravenously, during surgery Cortisone acetate = 25–37.5 mg/day orally Postsurgical hypocortisolism = 39 patients were retested at 6, 12, 18, 24, and 36 months by low-dose corticotropin stimulation test in 6, 15, 9, 7, and 2 patients.	N1 vs. N2 ACTH pg/mL: 9.6 ± 5.7 (1.9–26.9) vs. 9.0 ± 4.4 (5.0–23.8), *p* = 0.665 ACTH > 26.9 pg/mL: 0 (0.0) vs. 1 (1.7), *p* = 0.650 1 mg-DST (µg/dL): 4.6 ± 3.5 (1.2–13.7) vs. 3.2 ± 2.1 (0.9–9.0), *p* = 0.098 1 mg-DST < 1.2 µg/dL: 0 (0.0) vs. 9 (42.9), *p* < 0.0001 UFC (µg/24 h): 68.2 ± 45.2 (10.4–189.6) vs. 51.3 ± 32.4 (10.0–120.0), *p* = 0.136 UFC < 10.4 µg/24 h: 0 (0.0) vs. 2 (9.5), *p* = 0.119 MSC (µg/dL): 6.0 ± 3.1 (1.2–12.5) vs. 3.4 ± 2.0 (1.0–8.0), *p* = 0.001 MSC < 1.2 µg/dL: 0 (0.0) vs. 1 (4.8), *p* = 0.350 1 mg DST cortisol < 1.2 µg/dL had lower rates of dyslipidemia and a better metabolic profile
[33]	N1 vs. N2 Glucocorticoid replacement was lower in N1 < N2: 59% vs. 89%, *p* = 0.003 Treatment durations were shorter in N1 < N2: 4.4 ± 3.8 vs. 10.7 ± 18.0 months, *p* = 0.04	N1 vs. N2 Postoperative serum cortisol levels had greater values N1 > N2: 8.0 ± 5.7 vs. 5.0 ± 2.6 µg/dL, *p* = 0.03
[34]	N1 vs. N2 vs. N3 Postoperative length of hospital (days), median (IQR): 3 (2–5) vs. 3 (2–3) vs. 2 (2–4) Postoperative treatment due to adrenal insufficiency, postoperatively: 72.8% vs. 59.5% vs. 10.5% Postoperative treatment due to adrenal insufficiency, first follow-up: 71.9% vs. 50% vs. 14.4% Surgical technique open/endoscopic: 5.6%/94.4% vs. 8.9%/91.1% vs. 14.3/85.7	NA
[35]	Glucocorticoid replacement was started in patients who developed adrenal insufficiency. Prevalence of postoperative adrenal insufficiency = 25.9%	N1 vs. N2 At the time of diagnosis, both groups had similar cortisol metabolism. However, differences were observed after surgery: Basal cortisol (µg/dL): 12.8 (0.8–24.7) vs. 18.15 (12.1–25.5), *p* = 0.07 1 mg DST (µg/dL): 1.1 (0.5–8.7) vs. 2.9 (2.1–4.9), *p* = 0.003 ACTH (pg/mL): 20 (9–53) vs. 6 (1–9), *p* = 0.001 24 h UFC (nmol/24 h): 77 (28–121) vs. 85.5 (53–173), *p* = 0.41 Salivary cortisol (µg/dL): 0.15 (0.06–0.54) vs. 0.2 (0.09–0.31), *p* = 0.53 DHEA-S (µg/dL): 120 (15–129) vs. 72 (22–89), *p* = 0.62
[36]	N1 vs. N2 vs. N3 vs. N4 Need for postoperative glucocorticoid replacement: 57% vs. 74% vs. 5% vs. 50%, *p* = 0.001 Duration glucocorticoid replacement, months: 2.1 (0.8–4.6) vs. 6.0 (1.4–17.1) vs. 1 vs. 0.8 (0.7–2.2), *p* = 0.010 All patients who had abnormal POD1-CST were given replacement glucocorticoids. Hydrocortisone daily dose = 30 mg	N1 vs. N2 vs. N3 vs. N4 Abnormal POD1: 55% vs. 67% vs. 14% vs. 42%, *p* < 0.001 POD1-CST identifies patients with hypercortisolism who are at risk of adrenal insufficiency after unilateral adrenalectomy, allowing for selective glucocorticoid replacement.
[38]	Laparoscopic adrenalectomy = all patients There were no perioperative complications following surgery Hydrocortisone replacement = all patients, 2 months, and then adrenal function was regularly evaluated by corticotropin test Prevalence of adrenal insufficiency = 40% The mean duration of impaired adrenal function was 12.3 ± 9.0 (6–35) months	Follow-up: 6 months N1 vs. N2 ACTH (pg/mL): 26.0 ± 10.6 vs. 11.6 ± 4.9, *p* < 0.0001 1 mg DST (nmol/L): 19.3 ± 5.5 vs. 85.6 ± 27.6, *p* < 0.0001 UFC (nmol/24 h): 48.8 ± 27.6 vs. 60.2 ± 32.3, *p* = 0.22 UFC/UFC ratio: 0.22 ± 0.10 vs. 0.26 ± 0.15, *p* = 0.61 Patients with hypoadrenalism showed baseline 1 mg DST levels comparable to patients without (3.7 ± 0.7 vs. 3.0 ± 1, *p* = 0.107).
[39]	N1′ vs. N2′ vs. N3′ Duration of inpatient hospital stay (days): 6 ± 12 vs. 5 ± 20 vs. 5.5 ± 10, *p* = 0.465 Complications: 30% vs. 20% vs. 7.1%, *p* = 0.275 Postoperative adrenal insufficiency: 4.5% vs. 42.9% vs. 64.3%, *p* < 0.001 Duration of adrenal insufficiency (months): 47 vs. 4.5 ± 40 vs. 17 ± 130, *p* = 0.310 Minimally invasive surgery (N,%): 86.4% vs. 89.5% vs. 91.7%, *p* = 0.539 N’ Laparoscopic surgery = 71.7% Median postoperative follow-up = 72 (6–214) months. Low-grade postoperative complications = 20.4% Acute pancreatitis = 2 Allergic reaction = 4 Hypokalemia = 2 Hypoxia = 2 Intestinal atony = 2 Large hematoma = 2 Lymph fistula = 4 Pneumonia = 2 Wound infection = 2	NA
[40]	The laparotomic or laparoscopic method was chosen based on the size of the adrenal adenoma and the patient’s clinical features. Postoperative adrenal insufficiency = 81.2% (N = 26 of these N1 = 3, N2 = 23) Recovery within 6 weeks = 53.8% (N = 14 of these N1 = 3, N2 = 11) Recovery within 6 months = 16.6% (N2 = 2) Recovery within 1 year after surgery = 25% (N2 = 3) Without recovery of adrenal function at the end of follow-up = 26. 9% (N2 = 7) Hydrocortisone replacement = all patients Hydrocortisone daily dose = 25–37.5 mg	ROC curve identified a 1 mg DST test threshold of ≤4.7 µg/dL predicting 6-week recovery with 89.5% sensitivity and 72.7% specificity (area under curve 0.87; 95% CI 66.9–98.7, *p* < 0.001)-best predicted HPA axis recovery
[41]	Retroperitoneoscopic adrenalectomy = all patients (partial adrenalectomy = 92.7%, total adrenalectomy = 7.3%) Perioperative complication = one patient with type 2 diabetes mellitus had a delayed healing of the surgical incision. Follow-up: 12 months Glucocorticoid replacement was not necessary during the follow-up.	NA
[42]	N1 vs. N2 vs. N3 vs. N4 Received perioperative dexamethasone (4–10 mg): 36% vs. 32% vs. 27% vs. 33% Median days on glucocorticoid replacement (IQR): 246 (116–530) vs. 55 (36–133) vs. 81 (74–206) vs. 26 (15–36), *p* < 0.01 Patients with secondary adrenal insufficiency on CST received hydrocortisone at a standard dose of 20 mg in the morning and 10 mg in the evening.	N1 vs. N2 vs. N3 vs. N4 Median POD1 ACTH (pg/mL): 8.2 (3.6–29) vs. 12.4 (3.6–29.4) vs. 7.5 (5.6–10.5) vs. 13.8 (5.8–19.6), *p* = 0.316 Median POD1 basal cortisol (μg/dL): 5.0 (1.3 -12.3) vs. 8.5 (3.2–13.1) vs. 5.5 (1.6–13.3) vs. 11.4 (2.4–15.1), *p* = 0.256 Abnormal CST: 62% vs. 50% vs. 32% vs. 14%, *p* = 0.043 Using POD1 CST instead of basal cortisol levels alone helped identify people who were at risk for secondary adrenal insufficiency.
[43]	Minimally invasive adrenalectomy = all patients Hydrocortisone replacement = all patients Hydrocortisone daily dose = 50 mg of hydrocortisone 30 min before surgery, every 8 h on the same day, on the day after every 12 h, and then reduced to 25 mg every 12 h. Post-adrenalectomy adrenal insufficiency was assessed 5 days after surgery using a 250 μg corticotropin stimulation test, with peak cortisol levels ≤ 18 µg/dL. Prevalence of adrenal insufficiency = 67.4% There were no perioperative complications following surgery	NA
[44]	Laparoscopic unilateral adrenalectomy = all patients Hydrocortisone replacement = all patients Hydrocortisone daily dose = at discharge, patients received 20–30 mg/day, this dose was tapered by 2.5–5 mg/day every 2–4 weeks, and HPA returned to normal within 6 months after surgery There were no perioperative complications following surgery	NA

Abbreviations: ACTH = adrenocorticotropic hormone; CI = confidence interval; CST = cosyntropin stimulation test; DHEA-S = dehydroepiandrosterone sulphate; DST = dexamethasone suppression test; HPA = hypothalamic–pituitary–adrenal; IQR= interquartile range; MSC = midnight serum cortisol; NA = not available; N = number of patients; POD1 = postoperative day 1 cortisol; UFC = urinary free cortisol; vs. = versus, y = years (the study subgroups are presented in Table 1).

**Table A2:** Post-adrenalectomy impact on the cardiovascular and metabolic ailments in patients with MACS/NFA [34,35,37,38,39,41,43,44].

Reference	Impact of Adrenalectomy on Cardiovascular and Metabolic Morbidity in Patients with MACS or NFA
[34]	Median follow-up, N1 vs. N2 vs. N3: 3 (0–9.9) y vs. 2.6 (0–9.6) y vs. 3.2 (0–9.9) y Defined daily dose (DDD) was used to calculate drug consumption per patient-year, based on dispensed prescriptions the year before adrenalectomy and annually after adrenalectomy. N1 mean DDD difference (SD), *p*-value Hypertension: 198.4 (728.1), *p* = 0.003, increased medication at one year postoperatively Diabetes: 22.8 (184.7), *p* = 0.166 Hyperlipidemia: −59.4 (306.6), *p* = 0.013, decreased medication Antibiotics: 3.5 (47.9), *p* = 0.412 N2 mean DDD difference (SD), *p*-value Hypertension: 96.4 (598.4), *p* = 0.286 Diabetes: −10.6 (164.7), *p* = 0.668 Hyperlipidemia: 81.6 (389.2), *p* = 0.167 Antibiotics: −2.5 (22.5), *p* = 0.464 N3 mean DDD difference (SD), *p*-value Hypertension: 184.2 (510.0), *p* < 0.001, increased medication Diabetes: 0.624, *p* = 0.624 Hyperlipidaemia: 29.2 (268.3), *p* = 0.281 Antibiotics: 2.5 (19.8), *p* = 0.205
[35]	N1 vs. N2 Median duration of follow-up: 21 (2–126) months vs. 48 (10–88) months, *p* = 0.3 Diabetes mellitus: 40.7% vs. 37.5%, *p* = 0.602 Improvement of diabetes mellitus: 45% vs. 0, *p* = 0.12 Hypertension: 70.4% vs. 87.5%, *p* = 0.31 Improvement of hypertension: 68.4% vs. 0, *p* = 0.002 Hyperlipidemia: 29.6% vs. 37.5%, *p* = 0.49
[37]	The proportion of patients who initiated: N1′ vs. N1″ Antihypertensive treatment: 0% vs. 9.5%, *p* = 0.746 Hypoglycemic drugs: 0% vs. 4.7%, *p* = 0.586 Lipid-lowering medications: 16.7% vs. 13.6%, *p* = 0.833 N2′ vs. N2″ Antihypertensive treatment: 0% vs. 11.6%, *p* = 0.342 Hypoglycemic drugs: 0% vs. 6.8%, *p* = 0.315 Lipid-lowering medications: 36.4% vs. 22.4%, *p* = 0.317
[38]	N1′ vs. N2′ Improvement of blood pressure control: 68% vs. 13.4%, *p* = 0.001 Worsening of blood pressure control: 4% vs. 26.7, *p* = 0.03 The surgical approach was independently associated with the improvement of blood pressure control (OR = 3.0, 95%CI: 3.8–108.3, *p* < 0.001) Improvement of glycometabolic control: 28% vs. 3.3%, *p* = 0.02 Worsening of glycometabolic control: 8% vs. 20%, *p* = 0.12 Amelioration of blood pressure and/or glycometabolic control: 68% vs. 17%, *p* < 0.001 Worsening blood pressure and/or glycometabolic control: 40% vs. 12%, *p* = 0.03
[39]	Baseline, N′ vs. N″ Arterial hypertension: 77% vs. 74.9%, *p* = 0.731 Diabetes mellitus: 18% vs. 21.1%, *p* = 0.941 Dyslipidemia: 47.5% vs. 46.5%, *p* = 0.868 Obesity: 34.5% vs. 34.6%, *p* = 0.883 Last follow-up, N′ vs. N″ Arterial hypertension: 61.7% vs. 74.9%, *p* < 0.05 Diabetes mellitus: 26.2% vs. 30.2%, *p* = 0.691 Dyslipidemia: 44.3% vs. 53.8%, *p* = 0.442 Obesity: 35.6% vs. 38.5%, *p* = 0.195
[41]	One year after surgery Improvement in hypertension = 71 patients (57.7%) Blood pressure returned to normal levels without antihypertensive drugs = 21 patients (17.1%) Conservative management 10 patients (5.4%) complained about worsening of their blood pressure condition, and the type/dose of antihypertensive drugs was increased
[43]	Median follow-up: 48 (3–66) months N1 vs. N2 Improved Weight control: 32.6% vs. 6.5%, *p* = 0.002 Glucose control: 45.7% vs. 15.2%, *p* = 0.002 Blood pressure control: 45.7% vs. 23.9%, *p* = 0.029 Dyslipidemia control: 26.1% vs. 19.6%, *p* = 0.456 Worsened Weight control: 6.5% vs. 19.6%, *p* = 0.063 Glucose control: 10.9% vs. 43.5%, *p* < 0.001 Blood pressure control: 19.6% vs. 50%, *p* = 0.002 Dyslipidemia control: 34.8% vs. 47.8%, *p* = 0.204
[44]	Follow-up: 12 months N2′ vs. N2″ Overweight/obesity: 54.5% vs. 50%, *p* = 1.000 Impaired glucose regulation/Type 2 diabetes mellitus: 45.5% vs. 33.3%, *p* = 1.000 Hypertension: 45.5% vs. 66.7%, *p* = 0.620 Dyslipidemia: 63.6% vs. 83.3%, *p* = 0.600

Abbreviations: CI = confidence interval; DDD = defined daily dose; MACS = mild autonomous cortisol excess; N = number of patients; NFA = non-functioning adrenal tumours; OR = odds ratio; SD = standard deviation; vs. = versus; y = years (the study subgroups are presented in Table 1).

**Table A3:** Post-adrenalectomy bone status in subjects with MACS/NFA [31,34,44].

Reference	Impact of Adrenalectomy on Bone Status
[31]	Median follow-up: 37 (6–92) weeks Osteocalcin: mean differences of 8.18 ng/mL (SD = 6.74, *p* = 0.04) CTX: mean difference of 0.14 ng/mL (SD = 0.12, *p* = 0.05) PINP: mean difference of 1.78 µg/L (SD = 35.4, *p* = 0.75) Sclerostin: mean difference of 107 pg/mL (SD = 181, *p* = 0.24)
[34]	Median follow-up, N1 vs. N2 vs. N3: 3 (0–9.9) y vs. 2.6 (0–9.6) y vs. 3.2 (0–9.9) y. DDD was used to calculate drug consumption per patient-year, based on dispensed prescriptions the year before adrenalectomy and annually after adrenalectomy. N1 mean DDD difference (SD), *p*-value Osteoporosis: -14.3 (102.7), *p* = 0.119 N2 mean DDD difference (SD), *p*-value Osteoporosis: 7.5 (51.5), *p* = 0.336 N3 mean DDD difference (SD), *p*-value Osteoporosis: 5.7 (31.0), *p* = 0.072
[44]	Follow-up: 12 months N2′ vs. N2″ Osteopenia/osteoporosis: 63.6% vs. 50%, *p* = 0.644

Abbreviations: CTX = C-terminal telopeptide; DDD = defined daily dose; N = number of patients; SD = standard deviation; PINP = type 1 procollagen amino terminal peptide; vs. = versus (the study subgroups are presented in Table 1).

**Table A4:** Adrenalectomy and potential impact of the cognitive function in patients with MACS/NFA [34,44].

Reference	Impact of Adrenalectomy on the Cognitive Function: Outcomes
[34]	Median follow-up, N1 vs. N2 vs. N3: 3 (0–9.9) y vs. 2.6 (0–9.6) y vs. 3.2 (0–9.9) y. DDD was used to calculate drug consumption per patient-year, based on dispensed prescriptions the year before adrenalectomy and annually after adrenalectomy. N1 mean DDD difference (SD), *p*-value Depressive disease: −30.3 (226.7), *p* = 0.134 N2 mean DDD difference (SD), *p*-value Depressive disease: −47.6 (153.2), *p* = 0.043, decreased medication N3 mean DDD difference (SD), *p*-value Depressive disease: 4.1 (171.5), *p* = 0.814
[44]	Follow-up: 12 months N2′ vs. N2″ Global cognition (scores) Mini-Mental State Examination: 29.5 ± 0.9 vs. 28.5 ± 1.4, *p* = 0.726 Montreal Cognitive Assessment: 27.8 ± 1.8 vs. 26.2 ± 1.5, *p* = 0.062 Repeatable Battery for the Assessment of Neuropsychological Status: 107.0 ± 13.8 vs. 97.3 ± 10.6, *p* = 0.035 Visuospatial/constructional: 113.0 ± 9.2 vs. 96.2 ± 12.3, *p* = 0.033 Delayed memory: 104.1 ± 10.4 vs. 93.1 ± 14.5, *p* = 0.025 Immediate memory: 94.1 ± 18.1 vs. 84.3 ± 15.2, *p* =0.282 Language: 103.4 ± 11.0 vs. 100.5 ± 7.9, *p* = 0.666 Attention: 111.3 ± 9.7 vs. 117.7 ± 5.5, *p* = 0.211 Working memory: 14.8 ± 2.1 vs. 14.0 ± 2.0, *p* = 0.751 Executive function: 58.4 ± 23.9 vs. 59.6 ± 14.7, *p* = 0.579 Processing speed: 104.8 ± 62.6 vs. 75.2 ± 33.2, *p* = 0.596

Abbreviations: DDD = defined daily dose; N = number of patients; SD = standard deviation; vs. = versus; y = years (the study subgroups are presented in Table 1).
